# Supplementary material for: Evolutionary history and past climate change shape the distribution of genetic diversity in terrestrial mammals
Source: Nat Commun. 2020 May 22;11:2557. doi: 10.1038/s41467-020-16449-5 (PMC7244709; doi:10.1038/s41467-020-16449-5)
Supplement: Supplementary file 1 — Supplementary Information [file 41467_2020_16449_MOESM1_ESM.pdf]

Supplementary Information

Evolutionary history and past climate change shape the distribution of genetic diversity in terrestrial mammals

Theodoridis et al.

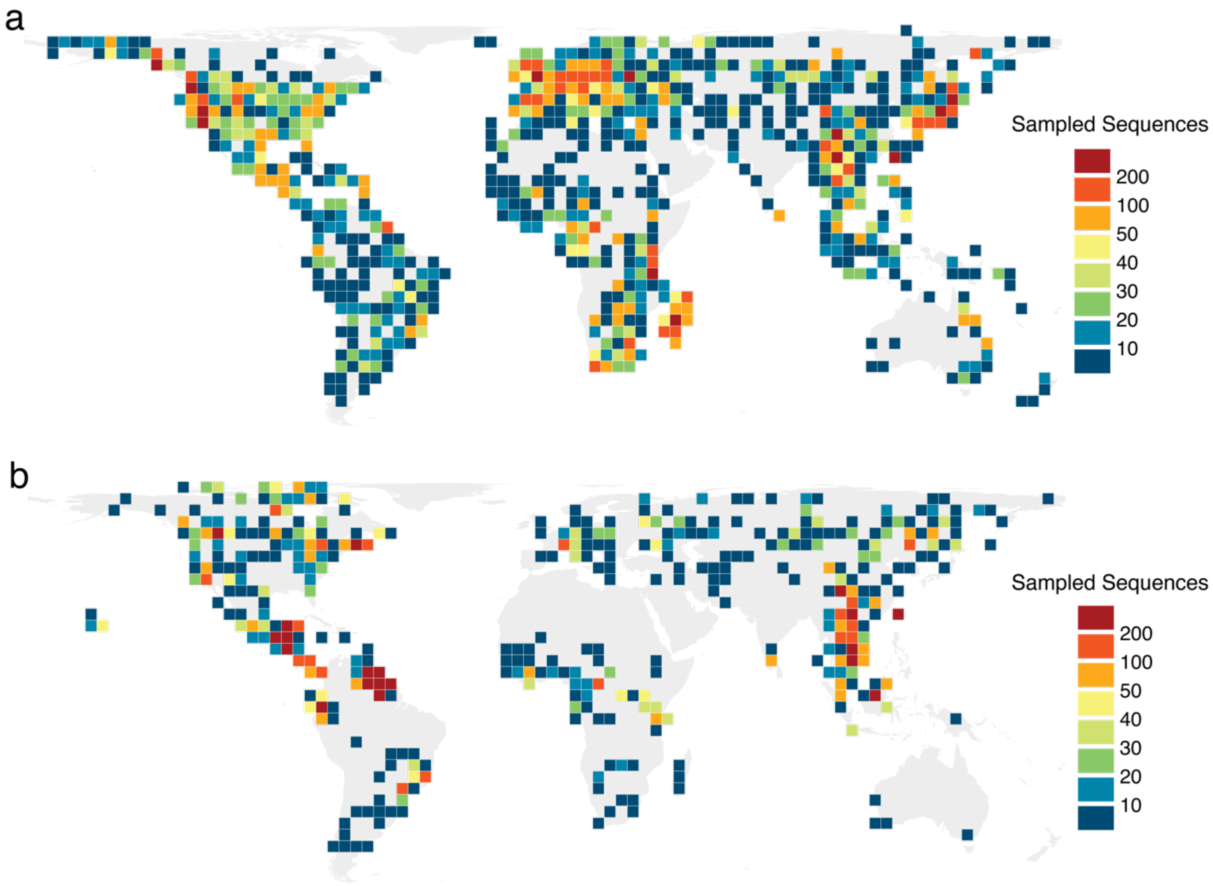

Supplementary Figure 1. Sampled genetic data for terrestrial mammals.

Number of available georeferenced sequences for *cytb* (a) and *co1* (b).

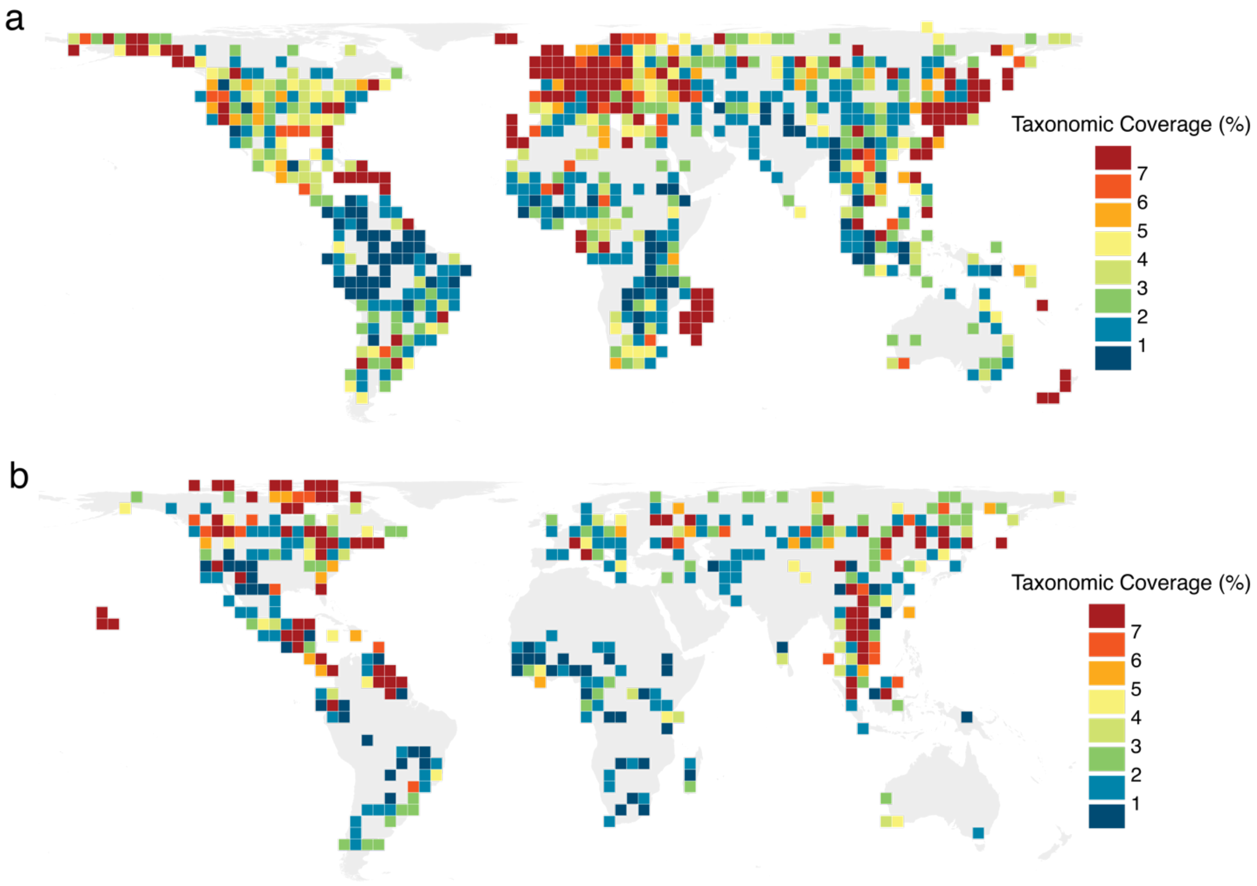

Supplementary Figure 2. Taxonomic coverage of genetic data for terrestrial mammals.

Taxonomic coverage (number of sampled species divided by species richness) for *cytb* (a) and *co1* (b).

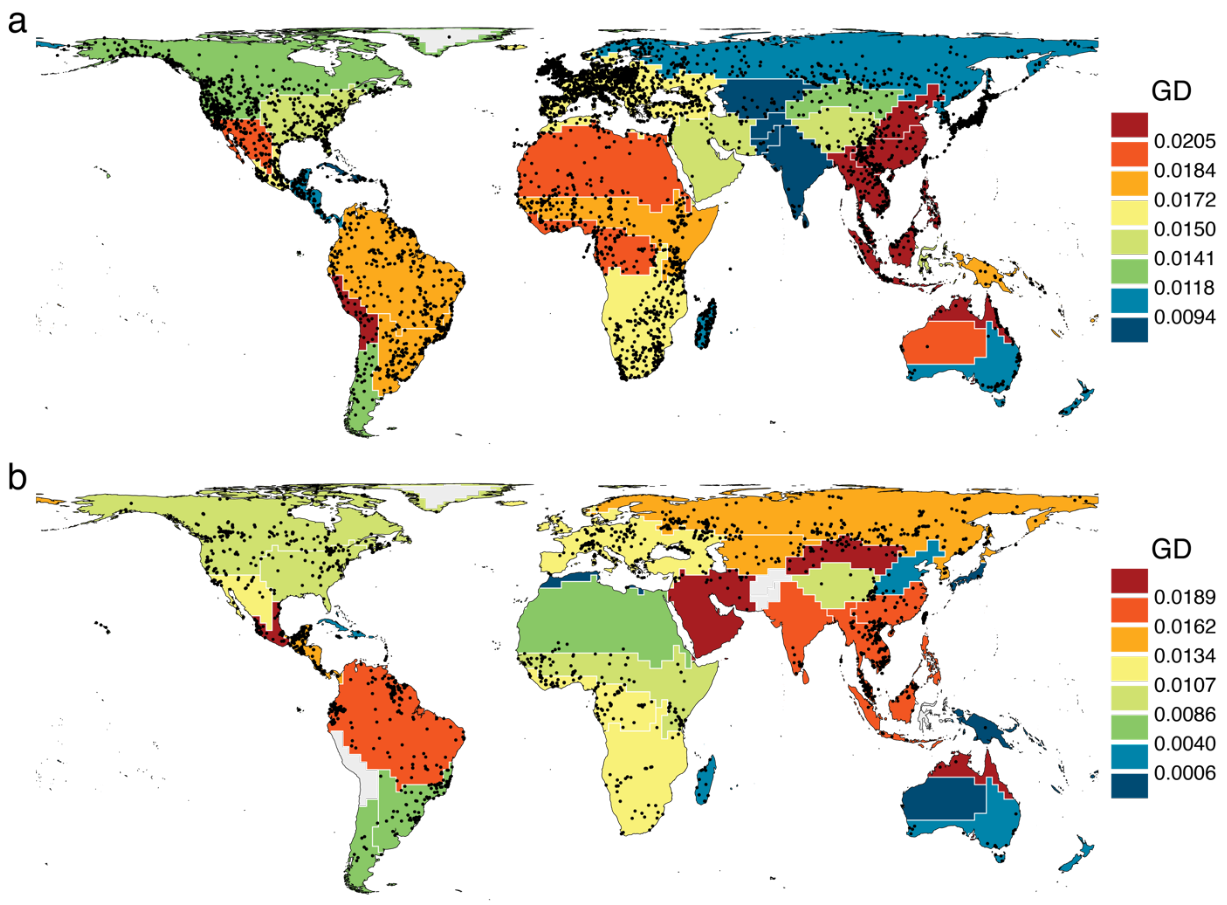

Supplementary Figure 3. Genetic diversity across zoogeographic regions for terrestrial mammals.

Genetic diversity (GD) for *cytb* (**a**) and *co1* (**b**) across the 34 zoogeographic regions for terrestrial mammals. Black dots represent the georeferenced sequences used for estimating GD within each region. Grey regions for *co1* represent regions without data.

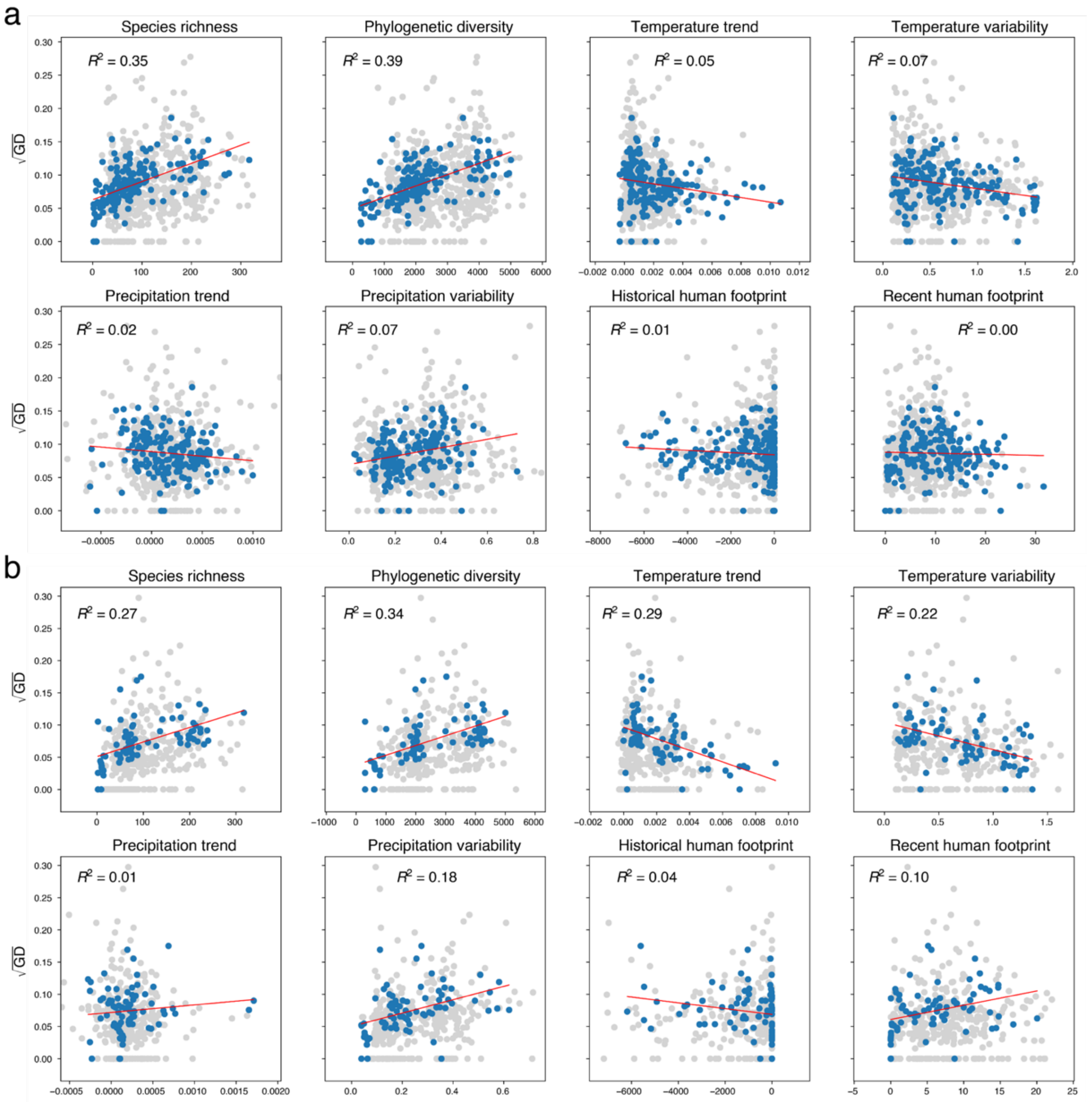

Supplementary Figure 4. Correlations between genetic diversity (GD) and eight independent variables and at the grid cell scale.

Linear regression models showing the correlation (R^2) between GD (square root) for *cytb* (**a**) and *co1* (**b**) and eight variables–that is, species richness, phylogenetic diversity, trend and variability in temperature and precipitation, and human footprint (historical and recent). Models are built for subsets (blue points; *cytb: n* = 185*; co1: n* = 76) of the full datasets (grey points) based on a minimum taxonomic coverage of 7% per cell and a minimum number of sequences per cell (55 sequences for *cytb*, 278 sequences for *co1*).

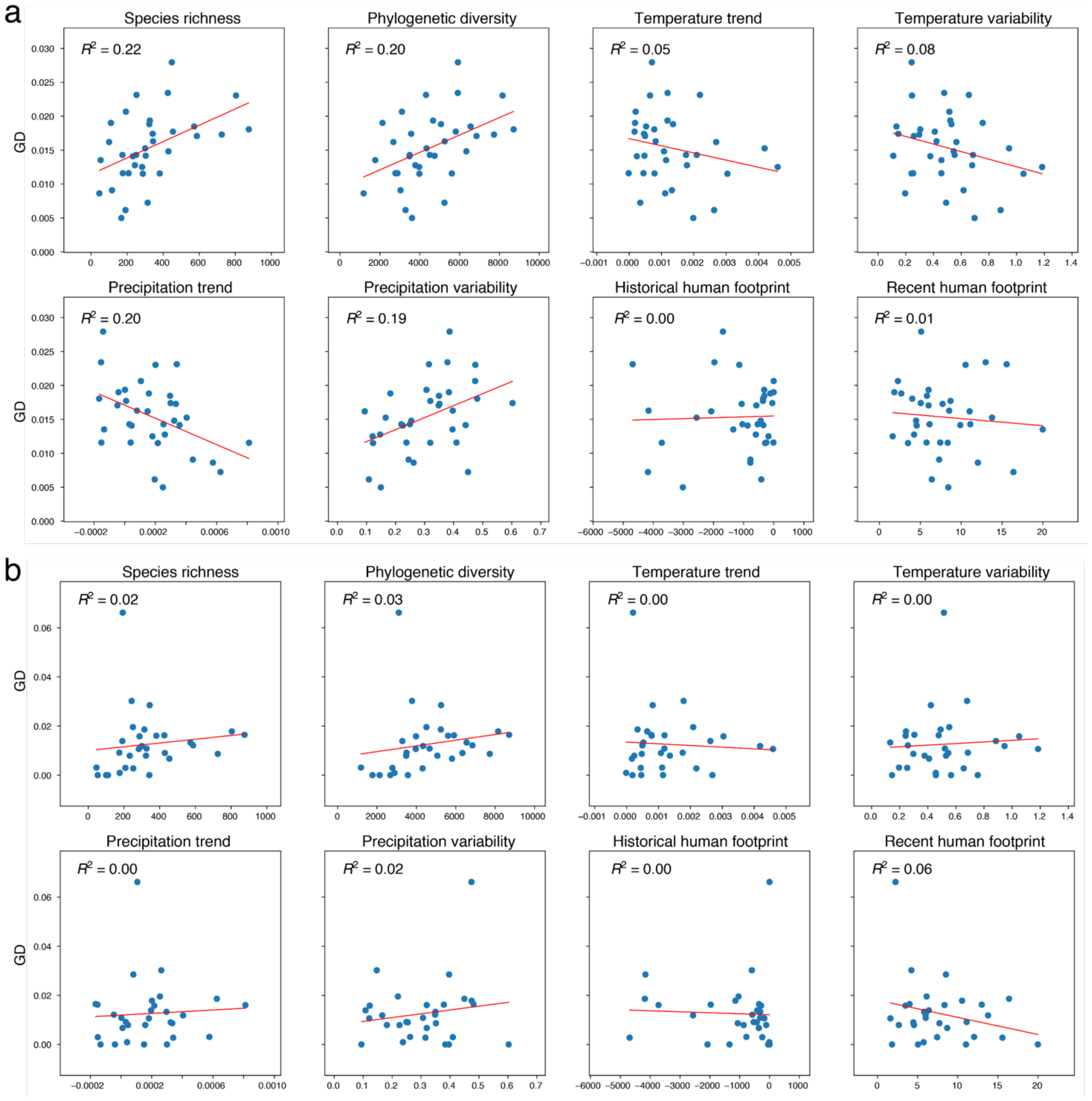

Supplementary Figure 5. Correlations between genetic diversity (GD) and eight independent variables and at the zoogeographic region scale.

Linear regression models showing the explained variance (R^2) in GD for *cytb* (**a**) (n = 34) and *co1* (**b**) (n = 30) by eight explanatory variables–that is, species richness, phylogenetic diversity, trend and variability in temperature and precipitation, and human footprint (historical and recent).

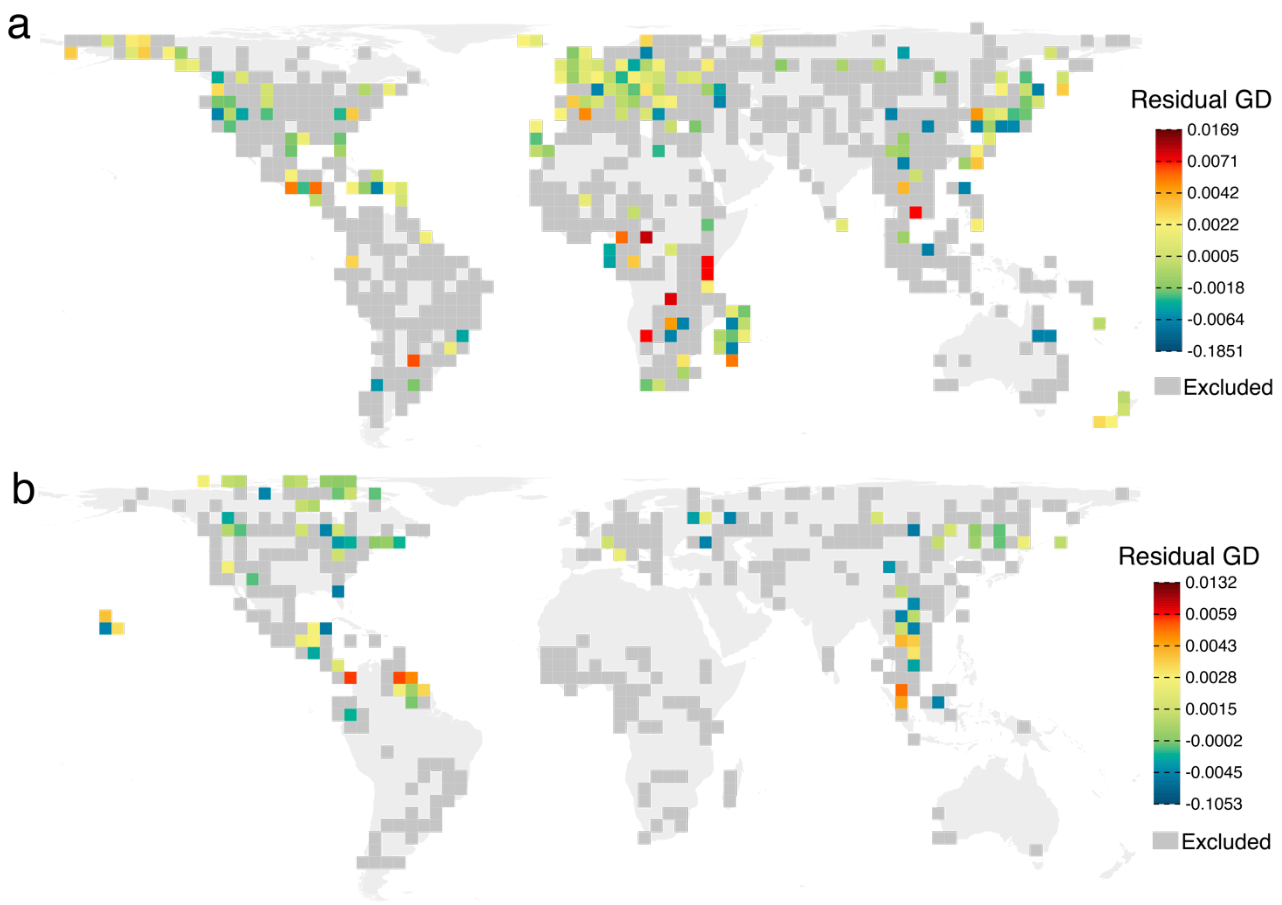

Supplementary Figure 6. Residual genetic diversity from model predictions.

Spatial distribution of residuals in predicted genetic diversity for the models presented in Fig. 3 (see also Tables 1 and 2) for *cytb* (**a**) and *co1* (**b**). Grey cells show the excluded cells based on the selection criteria per cell (that is, minimum taxonomic threshold, number of sequences).

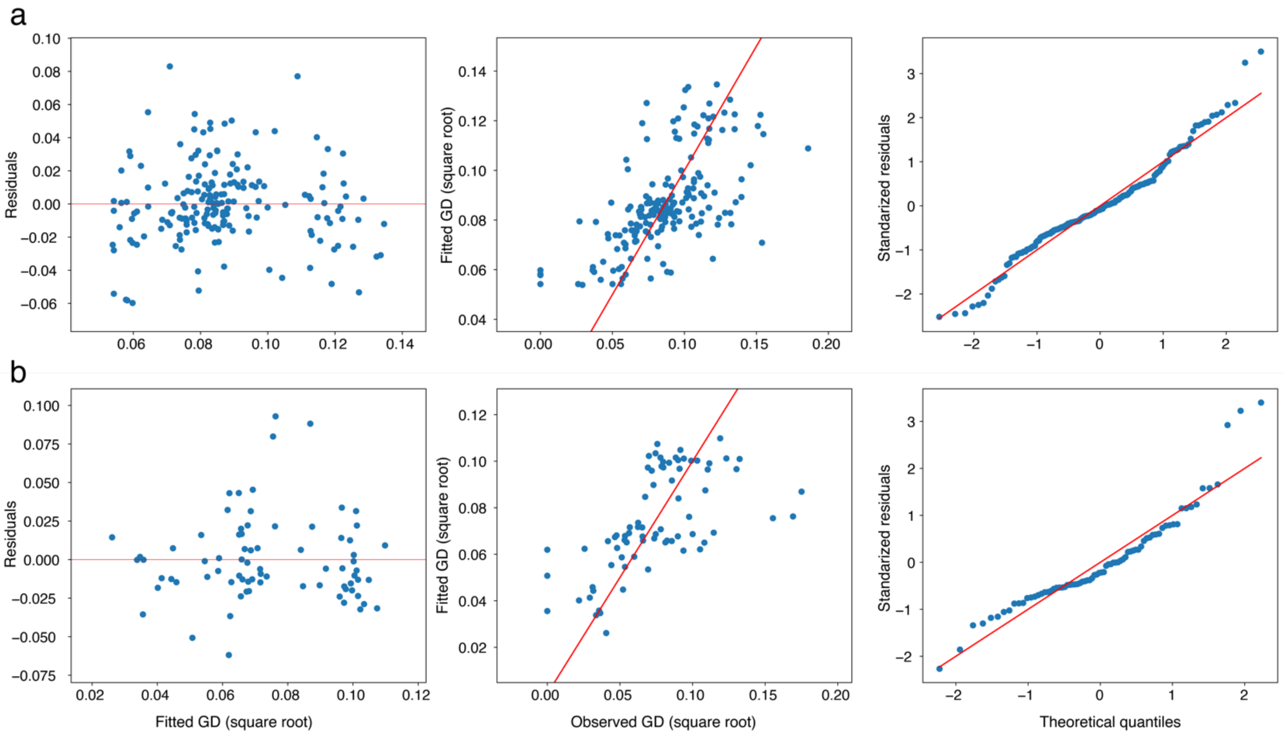

Supplementary Figure 7. Model diagnostics for predicted genetic diversity.

Diagnostic plots for the models presented in Fig. 3 and in Supplementary Figure 6 (see also Tables 1 and 2) for *cytb* (**a**) and *co1* (**b**).

Supplementary Table 1. Pairwise correlations between all eight independent variables at the grid cell scale.

	SR	PD	Temperature trend	Temperature variability	Precipitation trend	Precipitation variability	Historical human footprint	Recent human footprint
cytb								
SR	-	**0.98**	-0.37	-0.34	-0.13	0.38	-0.09	-0.17
PD	**0.98**	-	-0.33	-0.32	-0.11	00.36	-0.14	-0.13
Temperature trend	-0.37	-0.33	-	0.64	0.6	0.5	-0.44	0.19
Temperature variability	-0.34	-0.32	0.64	-	0.32	-0.56	-0.08	-0.19
Precipitation trend	-0.13	-0.11	0.6	0.32	-	-0.49	-0.39	0.12
Precipitation variability	0.38	0.36	-0.5	-0.56	-0.49	-	0.22	0
Historical human footprint	-0.09	-0.14	-0.44	-0.08	-0.39	0.22	-	-0.43
Recent human footprint	-0.17	-0.13	0.19	-0.19	0.12	0	-0.43	-
co1								
SR	-	**0.98**	-0.67	**-0.74**	0.27	**0.77**	-0.17	0.28
PD	**0.98**	-	-0.68	**-0.75**	0.32	**0.75**	-0.26	0.35
Temperature trend	-0.67	-0.68	-	**0.76**	-0.12	**-0.71**	0.13	-0.37
Temperature variability	**-0.74**	**-0.75**	**0.76**	-	-0.24	**-0.82**	0.28	-0.36
Precipitation trend	0.27	00.32	-0.12	-0.24	-	0.29	-0.16	0.09
Precipitation variability	**0.77**	**0.75**	**-0.71**	**-0.82**	0.29	-	-0.12	0.36
Historical human footprint	-0.17	-0.26	0.13	0.28	-0.16	-0.12	-	-0.39
Recent human footprint	0.28	0.35	-0.37	-0.36	0.09	00.36	-0.39	-

Values correspond to Pearson correlation coefficient. Sample size $n = 185$ for *cytb* and $n = 76$ for *co1*. Bold values indicate high correlation (Pearson's $|r| > 0.7$). Models that included highly correlated variables were not evaluated in the multimodel inference (see Methods).

Supplementary Table 2. Pairwise correlations between all eight independent variables at the zoogeographic region scale.

	**SR**	**PD**	**Temperature trend**	**Temperature variability**	**Precipitation trend**	**Precipitation variability**	**Historical human footprint**	**Recent human footprint**
SR	-	**0.96**	-0.25	-0.39	-0.11	0.41	0.04	-0.16
PD	**0.96**	-	-0.2	-0.33	-0.04	0.32	-0.1	-0.11
Temperature trend	-0.25	-0.2	-	**0.79**	0.17	**-0.7**	-0.14	0.06
Temperature variability	-0.39	-0.33	**0.79**	-	0	-0.68	-0.02	-0.12
Precipitation trend	-0.11	-0.04	0.17	0	-	-0.15	-0.38	0.19
Precipitation variability	0.41	0.32	**-0.7**	-0.68	-0.15	-	-0.02	0.14
Historical human footprint	0.04	-0.1	-0.14	-0.02	-0.38	-0.02	-	-0.6
Recent human footprint	0.16	0.11	0.06	-0.12	0.19	0.14	-0.6	-

Values correspond to Pearson correlation coefficient. Sample size $n = 34$. Bold values indicate high correlation (Pearson's $|r| > 0.7$). Models that included highly correlated variables were not evaluated in the multimodel inference (see Methods).

Supplementary Table 3. Modified t-test (two-sided) and *P*-values for the significance of the spatial association between GD for *cytb* and PD and SR across subsets of our full data set based on minimum taxonomic threshold (see also Fig. 2).

		**Biodiversity Variables**			
		**Phylogenetic Diversity**		**Species Richness**	
Minimum taxonomic threshold (%)	Sample size (n of cells)	modified t-test	*P*-value	modified t-test	*P*-value
0	717	0.234	**0.0001**	0.225	**0.0001**
1	637	0.275	**0**	0.265	**0**
2	457	0.362	**0**	0.351	**0**
3	355	0.421	**0**	0.402	**0**
4	277	0.484	**0**	0.454	**0**
5	228	0.552	**0**	0.525	**0**
6	203	0.571	**0**	0.542	**0**
7	185	0.621	**0**	0.59	**0**
8	167	0.628	**0**	0.595	**0**
9	158	0.635	**0**	0.602	**0**
10	152	0.627	**0**	0.596	**0**
11	146	0.628	**0**	0.594	**0**
12	144	0.62	**0**	0.587	**0**
13	140	0.604	**0.0001**	0.574	**0.0001**
14	139	0.603	**0.0001**	0.572	**0.0001**
15	132	0.58	**0.0003**	0.553	**0.0004**
16	129	0.625	**0.0001**	0.592	**0.0002**
17	129	0.625	**0.0001**	0.592	**0.0002**
18	129	0.625	**0.0001**	0.592	**0.0002**
19	127	0.628	**0.0001**	0.595	**0.0001**
20	127	0.628	**0.0001**	0.595	**0.0001**

Significant *P*-values (< 0.05) are highlighted in bold text. *P*-values correspond to a two-sided modified t-test.

Supplementary Table 4. Modified t-test and *P*-values for the significance of the spatial association between GD for *co1* and PD and SR across subsets of our full data set based on minimum taxonomic threshold (see also Fig. 2).

		**Biodiversity Variables**			
		**Phylogenetic Diversity**		**Species Richness**	
Minimum taxonomic threshold (%)	Sample size (n of cells)	modified t-test	*P*-value	modified t-test	*P*-value
0	391	0.268	**0.0001**	0.25	**0.0001**
1	331	0.332	**0**	0.314	**0**
2	217	0.432	**0**	0.405	**0.0001**
3	157	0.451	**0.0002**	0.414	**0.0005**
4	135	0.446	**0.0007**	0.402	**0.0019**
5	114	0.449	**0.0052**	0.403	**0.0109**
6	96	0.533	**0.0122**	0.471	**0.0268**
7	76	0.584	**0.0325**	0.522	0.0546
8	64	0.587	**0.0377**	0.521	0.0616
9	59	0.598	0.0603	0.522	0.0985
10	55	0.594	0.063	0.515	0.1062
11	45	0.577	0.0603	0.491	0.1083
12	35	0.566	0.1133	0.477	0.172
13	33	0.642	0.0872	0.57	0.1185
14	32	0.735	**0.0489**	0.709	0.0506
15	29	0.718	**0.0301**	0.69	**0.0333**
16	28	0.716	**0.0331**	0.687	**0.0366**
17	25	0.715	**0.0321**	0.682	**0.0323**
18	24	0.718	**0.036**	0.683	**0.037**
19	23	0.693	0.0859	0.659	0.084
20	22	0.701	0.0921	0.665	0.0906

Significant *P*-values (< 0.05) are highlighted in bold text. *P*-values correspond to a two-sided modified t-test.

Supplementary Table 5. Results of the iterative hierarchical partitioning approach.

	Spatial scale					
	Grid cells			Zoogeographic regions		
	IC (%)	Observed IC	Z score	IC (%)	Observed IC	Z score
cytb						
Species richness	40.0	0.16	18.65	-	-	-
Phylogenetic diversity	49.54	0.2	23.31	34.25	0.15	3.48
Temperature trend	-	-	-	-	-	-
Temperature variability	5.66	0.02	2.2	-	-	-
Precipitation trend	-	-	-	37.93	0.17	3.28
Precipitation variability	4.78	0.02	1.66	27.81	0.12	2.23
Historical human footprint	-	-	-	-	-	-
Recent human footprint	-	-	-	-	-	-
coI						
Species richness	26.04	0.12	6.02	-	-	-
Phylogenetic diversity	37.93	0.17	9.08	-	-	-
Temperature trend	23.28	0.11	4.84	-	-	-
Temperature variability	12.73	0.06	2.42	-	-	-
Precipitation trend	-	-	-	-	-	-
Precipitation variability	-	-	-	-	-	-
Historical human footprint	-	-	-	-	-	-
Recent human footprint	-	-	-	-	-	-

The independent contribution (IC) values correspond to the percentage of the explained variance in genetic diversity accounted for by each explanatory variable after excluding non-significant variables. Observed IC values for each variable and Z-scores for the generated distribution of randomized IC. Z-scores are calculated as (observed - mean(randomizations))/sd(randomizations), and statistical significance is based on the upper 0.95 confidence limit ($Z >= 1.65$).

Supplementary Table 6. Standardized and unstandardized regression coefficients for retained models of the multimodel inference (see also Tables 1 and 2).

	Regression coefficients (standardized / unstandardized)					
**Model Rank**	Phylogenetic diversity	Species richness	Temperature trend	Temperature variability	Precipitation trend	Precipitation variability
cytb						
**Grid cells**						
1	0.62 / 1.7e-5	-	-	-	-	-
2	0.6 / 1.63e-5	-	-	-0.076 / -5.8e-3	-	-
3	0.6 / 1.66e-5	-	-	-	-	0.04 / 9.9e-3
4	0.6 / 1.63e-5	-	-	-0.074 / -5.7e-3	-	0.002 / 5.4e-4
**Zoogeographic regions**						
1	0.35 / 9.96e-7	-	-	-	-0.39 / -8.53	0.26 / 0.011
2	0.43 / 1.24e-6	-	-	-	- 0.43 / -9.35	-
3	-	-	-	-	-0.38 / -8.44	0.38 / 0.015
co1						
**Grid cells**						
1	0.41 / 1.06e-5	-	-0.26 / -4.33	-	-	-
2	0.58 / 1.51e-5	-		-	-	-
3	-	0.3 / 1.28e-4	-0.34 / -5.66	-	-	-

Supplementary Note 1

Functionality of the web application https://geneticgeography.com/

==================

The web application will work only with modern versions of Google Chrome, Safari or Opera.

It facilitates the following actions:

- Interactive visual exploration of the spatial patterns of mammalian genetic diversity across spatial scales and genetic markers

- Interactive visual exploration of the relationships between major explanatory variables and genetic diversity

- Retrieval of the data used in the manuscript

Primary and secondary maps

The primary (bigger) map shows the distribution of genetic diversity in terrestrial mammals, while the secondary (smaller) map shows the distribution of the selected independent / explanatory variable. The user can zoom in and out using the plus and minus symbols at the top left of each map, and additionally drag the map to the desired location using the mouse.

Scatter plot panel

In the scatter plot the user can explore simple statistical relationships (simple linear regressions) between any of the included explanatory variables evaluated in the manuscript, i.e. phylogenetic diversity, species richness, climate stability (trend and variability in temperature and precipitation), and human footprint (historical and recent). The response variable is either the genetic diversity or its square root transformation. The conditional distribution of the variables is shown as histograms along the plot axes. Basic information about the selected dataset and the statistical relationships is shown in the upper left side of the plot.

Input data and filters

There are three sets of parameters in the Input Data and Filters container (below the primary map). Please note that in order for the filters to take effect after they are modified, the user must click the highlighted (in red) “Update” button in the “Actions” section.

- The first set of parameters in the “Data set” section defines the main dataset to be explored. The user can select the genetic marker (*cytb* or *co1*) and the spatial scale in which the genetic diversity of the selected marker was calculated

- The second set of parameters in the “Subset” section subsets the main dataset according to a minimum taxonomic coverage and a minimum number of sequences per spatial unit. Between the two control sliders the user can choose how to combine the two filters. The default value is the Boolean selector “or” that will select spatial units that pass either of the two filters, while the Boolean selector “and” will jointly evaluate the two filters and select spatial units that pass both

filters. When the user selects the grid scale from the “Data set” section, a red vertical line will appear in both sliders. Setting the minimum number of sequences and the minimum taxonomic coverage to the red line, the program will plot the dataset used for the statistical inferences in the manuscript

- The third set of parameters in the “Variables” section allows the user to select which variables to plot. The “Response” variable can only be the genetic diversity (or the square root of it) and will be plotted in the main map, while the “Independent” or explanatory variable will be plotted in the secondary map.

Finally, in the “Actions” section the user can select a dataset and apply the filters using the “Update” button. Additionally, the user can download the data that correspond to the main dataset (not the subsets) either as .csv or .json format. All the sequence accession numbers, the corresponding species, and the attached coordinates used in the manuscript (regardless of the spatial scale) can be downloaded by clicking the “Get sequences” button.

Information panel

By clicking on any spatial unit either in the maps or in the scatter plot panel, summary information for the respective unit appears in the “Spatial unit summary” section. Additionally, specific information for each taxonomic order within the spatial unit appears in the “Genetic diversity and sampled species per mammalian order” section.
