# Supplementary material for: Evolutionary history and past climate change shape the distribution of genetic diversity in terrestrial mammals
Source: Nat Commun. 2020 May 22;11:2557. doi: 10.1038/s41467-020-16449-5 (PMC7244709; doi:10.1038/s41467-020-16449-5)
Supplement: Supplementary file 3 — Reporting Summary [file 41467_2020_16449_MOESM3_ESM.pdf]

nature research

Corresponding author(s): Spyros Theodoridis
David Nogues-Bravo

Last updated by author(s): Apr 27, 2020

Reporting Summary

Nature Research wishes to improve the reproducibility of the work that we publish. This form provides structure for consistency and transparency in reporting. For further information on Nature Research policies, see Authors & Referees and the Editorial Policy Checklist .

Statistics

For all statistical analyses, confirm that the following items are present in the figure legend, table legend, main text, or Methods section.

n/a	Confirmed	
☐	☒	The exact sample size (*n*) for each experimental group/condition, given as a discrete number and unit of measurement
☒	☐	A statement on whether measurements were taken from distinct samples or whether the same sample was measured repeatedly
☐	☒	The statistical test(s) used AND whether they are one- or two-sided<br>*Only common tests should be described solely by name; describe more complex techniques in the Methods section.*
☐	☒	A description of all covariates tested
☐	☒	A description of any assumptions or corrections, such as tests of normality and adjustment for multiple comparisons
☐	☒	A full description of the statistical parameters including central tendency (e.g. means) or other basic estimates (e.g. regression coefficient) AND variation (e.g. standard deviation) or associated estimates of uncertainty (e.g. confidence intervals)
☐	☒	For null hypothesis testing, the test statistic (e.g. F, t, r) with confidence intervals, effect sizes, degrees of freedom and P value noted<br>*Give P values as exact values whenever suitable.*
☒	☐	For Bayesian analysis, information on the choice of priors and Markov chain Monte Carlo settings
☒	☐	For hierarchical and complex designs, identification of the appropriate level for tests and full reporting of outcomes
☒	☐	Estimates of effect sizes (e.g. Cohen's *d*, Pearson's *r*), indicating how they were calculated

Our web collection on statistics for biologists contains articles on many of the points above.

Software and code

Policy information about availability of computer code

Data collection	The retrieval and georeferencing of genetic sequences was conducted using Bash v4.4, api.geonames.org, Entrez Programming Utilities, and BOLD Public Data API
Data analysis	All analyses have been conducted with Geneious v8.1.7, MUSCLE v3.8.31, Python v3.7.5, and R v3.6.3. Aditional custom scripts are avialiable in https://github.com/spyrostheodoridis/Genetic-geography-of-terrestrial-mammals

For manuscripts utilizing custom algorithms or software that are central to the research but not yet described in published literature, software must be made available to editors/reviewers. We strongly encourage code deposition in a community repository (e.g. GitHub). See the Nature Research guidelines for submitting code & software for further information.

Data

Policy information about availability of data

All manuscripts must include a data availability statement. This statement should provide the following information, where applicable:

- Accession codes, unique identifiers, or web links for publicly available datasets
- A list of figures that have associated raw data
- A description of any restrictions on data availability

The identifiers for all genetic sequences used in this study and the processed data to recreate the figures are available in https://github.com/spyrostheodoridis/Genetic-geography-of-terrestrial-mammals. An interactive exploration of the reported findings is available through the web application https://geneticgeography.com. The Supplementary Note 1 provides instructions on the functionality of the web application. The raw genetic sequences are available in GeneBank (www.ncbi.nlm.nih.gov/genbank) and BOLD (www.boldsystems.org). Data used for the analysis of past climates are available through the PaleoView software (https://github.com/GlobalEcologyLab/PaleoView). Species range maps are available through IUCN (https://www.iucnredlist.org/resources/spatial-data-download). Dated phylogenies are available in Dryad (https://datadryad.org/stash/dataset/doi:10.5061/dryad.bp26v20). The anthropogenic land cover change during Holocene is available in PANGAEA (https://doi.pangaea.de/10.1594/PANGAEA.871369). The human footprint maps are available in Dryad (https://datadryad.org/stash/dataset/doi:10.5061/dryad.052q5). Data for zoogeographic regions are available in https://macroecology.ku.dk/resources/wallace.

Field-specific reporting

Please select the one below that is the best fit for your research. If you are not sure, read the appropriate sections before making your selection.

☐ Life sciences ☐ Behavioural & social sciences ☒ Ecological, evolutionary & environmental sciences

For a reference copy of the document with all sections, see nature.com/documents/nr-reporting-summary-flat.pdf

Ecological, evolutionary & environmental sciences study design

All studies must disclose on these points even when the disclosure is negative.

Study description	The study investigated the statistical associations between genetic diversity in terrestrial mammals and hypothesized covariates, such as phylogenetic diversity, past climate stability and land-use change. Genetic diversity was estimated within each spatial unit, i.e., grid-cell or zoogeographic region, using georeferenced genetic sequences.
Research sample	All available terrestrial mammal sequences for two mitochondrial genes , i.e., cytb and co1, were retrieved from public databases (GeneBank and BOLD). Due to their advantageous properties, these two genetic markers have been extensively used in taxonomic, phylogenetic and phylogeographic studies, and therefore constitute the richest resource of genetic data with available spatial information.
Sampling strategy	The sampling strategy aimed at maximizing the georeferencing of all available cytb and co1 sequences at the time of retrieval. The final sample size is 46,965 sequences. Several subsets of the full data set were further analyzed based on the data availability (number of sequences and taxonomic coverage) per spatial unit.
Data collection	Sen Li downloaded the genetic data from GeneBank and BOLD.
Timing and spatial scale	GeneBank data were downloaded on the 16th of May 2017. Data from BOLD were downloaded on the 2Sth of May 2017.
Data exclusions	We only kept sequences that had coordinates attached or those that included locality information below the country level. Georeferenced sequences falling outside of the respective species range by more than a predefined (385.9 km) distance threshold were excluded. Species with only one available sequence were also excluded.
Reproducibility	All statistical analyses were repeated >3 times and, where applicable, using alternative software to ensure maximum reproducibility.
Randomization	The study did not conduct any experiments for data collection.
Blinding	The study did not conduct any experiments for data collection.

Did the study involve field work? ☐ Yes ☒ No

Reporting for specific materials, systems and methods

We require information from authors about some types of materials, experimental systems and methods used in many studies. Here, indicate whether each material, system or method listed is relevant to your study. If you are not sure if a list item applies to your research, read the appropriate section before selecting a response.

Materials & experimental systems

n/a	Involved in the study	
☒	☐	Antibodies
☒	☐	Eukaryotic cell lines
☒	☐	Palaeontology
☒	☐	Animals and other organisms
☒	☐	Human research participants
☒	☐	Clinical data

Methods

n/a	Involved in the study	
☒	☐	ChIP-seq
☒	☐	Flow cytometry
☒	☐	MRI-based neuroimaging
